# LINKING JOB CHARACTERISTICS TO AMBULATORY DISABILITY BY RACE/ETHNICITY AND NATIVITY AMONG THE AGING WORKFORCE

**DOI:** 10.1093/geroni/igae098.0976

**Published:** 2024-12-31

**Authors:** Sung Park

**Affiliations:** University of Massachusetts Boston, Boston, Massachusetts, United States

## Abstract

Americans today are working longer, and more than half of adults aged 50+ work in physically demanding jobs. There are also well-known occupational risk factors for work injuries: physically demanding activities (PD) and nonstandard work schedules (NSW). This project uses a nationally representative, longitudinal sample, the Survey of Income and Program Participation, linked to a nationally representative dataset of objective, rather than subjective, measures of the work environment for all civilian occupations in the U.S. to examine the potential health burden of PD and NSW as sources of short- and longer-term ambulatory disability (AD) for older workers. Specifically, the study investigates whether the risks associated with working in jobs with NSW and high PD interact to produce risks in short- and longer-term AD that are larger than each risk acting independently. Moreover, this study includes an examination of differential exposure and differential vulnerability by race/ethnicity and nativity to these work characteristics on the risks of AD. Results suggest significant heterogeneity in occupational exposure to PD and NSW, with racial/ethnic minorities, especially female immigrants, at a particular disadvantage. Moreover, the associations between these work exposures with both short- and longer-term AD also vary dramatically by race/ethnicity, nativity, and gender. The findings advance our understanding of how work demands and organizational factors impact the physical health of the diversifying older workforce, and contribute to health disparities in later life.

